# How a co-actor’s (Un-) reliability modulates goal selection in a novel joint goal-setting paradigm

**DOI:** 10.1007/s00426-024-02056-2

**Published:** 2024-11-15

**Authors:** Felix J. Götz, Gesine Dreisbach

**Affiliations:** Institute of Psychology, University of Regensburg, Universitätsstr. 31, 93053 Regensburg, Germany

## Abstract

Sociomotor theory – an extension of ideomotor theory – suggests that actions can also be represented in terms of the effects they elicit from others. But what if those others violate one’s action effect anticipations? Here, we introduce a novel joint goal-setting paradigm to investigate effects of co-actors’ occasional *and* overall unreliability on an individual’s goal selection. In a first step, the participant moved a target halfway from the bottom center to the top left or right corner of the computer screen. In the second step, the co-actor moved the target to its final left or right position. In a learning block, the co-actor always continued the participant’s target movements. In the test block(s), the co-actor produced congruent action effects in 50% (unreliable) vs. 80% (reliable co-actor) of the trials. Experiment 1 consisted of one (between-participants), Experiment 2 and 3 of two (within-participants) test blocks; in Experiment 3, the co-actor changed between blocks. Results of Experiments 1 and 3 reveal that participants repeated their corner choice more often after incongruent trials, but only when the co-actor was generally reliable. Implications in terms of sociomotor action control and joint action are discussed.

Our actions have more or less predictable effects in the environment. Ideomotor theories suggest that goal directed action selection is guided by its anticipated (desired that is) effect in the environment (Harleß, 1861; Herbart, 1825; Hommel, 2009; James, 1890; cf. Stock & Stock, 2004). People press a button to ring a bell or operate a switch to turn on the lights. However, whether or not an action is followed by the anticipated effect is not always under the sole control of the actor, especially in joint-action settings (Sebanz et al., 2006; Vesper et al., 2010). For example, to hang a picture on the wall, the help of a co-actor is needed. While the actor monitors the position (higher or lower? ) and orientation (straight or crooked? ) of the picture and gives instructions when necessary (e.g. higher on the left! ), the co-actor is needed to realize these instructions. As long as the co-actor follows the instructions, there is no additional need to act. But what if the co-actor does not comply with the instructions? Moreover, what if the actor at some point feels that they can no longer rely on the co-actor to follow? In the present research, we introduce a novel experimental paradigm to investigate (1) how action selection is modulated by a co-actor’s action outcome. In addition, (2) we aim to investigate the influence of the co-actor’s overall reliability on action selection.

In the action-effect literature, two paradigms can be differentiated: Action-effect compatibility paradigms and action-effect acquisition paradigms (cf. Pfister, 2019). In *action-effect compatibility paradigms*, a free choice spatial key press (left, right) is facilitated when consistently followed by a spatially corresponding effect (Ansorge, 2002; Kunde, 2001; Pfister et al., 2014b; Pfister & Kunde, 2013). In *action-effect acquisition paradigms*, participants learn systematic action-effect associations in a learning phase, e.g., a left key press is always followed by high pitch tone, a right key press by a low pitch tone. Once this association is learned, participants are faster to respond to a high pitch tone with a left key press and to a low pitch tone with a right key press; when asked to freely choose a left or right key in response to high and low pitch tones, participants choose the formerly learned association more often. That is, response times and response choice depend on the learnt action-effect associations. (e.g., Elsner & Hommel, 2001; Pfister et al., 2011). One widespread explanation for these action-effect phenomena is the notion of a bidirectional link between motor actions and their sensory consequences, which allows the reciprocal activation of certain motor patterns by the images of the intended effects (ideomotor approach; e.g., Hommel, 2009; James, 1890). In contrast to both types of paradigms, though, we are interested here in the effects of *occasional violations* of anticipated action-effect couplings in social/joint action-effect settings (cf. Pfister et al., 2020).

Translating the ideomotor approach from individual to social settings, the sociomotor approach suggests that people can represent action effects not only in the inanimate environment, but also in terms of the behavior they elicit from others (Kunde et al., 2018).^1^ For example, studies on imitation showed that it is easier to produce to-be-imitated than to-be-counter-imitated actions (Pfister et al., 2013; see also Lelonkiewicz et al., 2020; Weller et al., 2020). Other studies replicated the performance benefits of action-effect associations in joint action settings, where participants press a button to trigger a specific action of another person, who in turn produces a visible effect in the environment (e.g., Müller, 2016, 2020 for a recent review, see Neszmélyi et al., 2022). Here, however, we are interested in sociomotor effects on *action selection* rather than performance (cf. Gaschler & Nattkemper, 2012).

The present research investigated as to how occasional violations of social action-effect anticipation affect the actor’s subsequent action selection. If post-response effects are anticipated for response production, the unrealized internal outcome representation should exert a continued effect on the motor system that presumably aims at its delayed manifestation in the (inanimate or social) environment (cf. Kunde et al., 2004; Prinz, 1997). In terms of the sociomotor approach (Kunde et al., 2018), repeating one’s goal selection is the most likely candidate for seeing one’s representation of the co-actor’s actions manifest eventually (see also joint action variants of predictive coding, Pesquita et al., 2018; Wolpert et al., 2003). Therefore, we predict that if a co-actor’s action outcome violates the actor’s action effect anticipation, the latter will tend to repeat their goal selection (i.e., show a *goal repetition effect*) in order to manipulate the co-actor’s subsequent action accordingly. This prediction, however, relies on the experience that the co-actor is reliable and follows the actor’s lead. Therefore, we always started with a learning phase, in which the co-actor consistently followed the participant’s lead (and thus fulfilled their goal). Only then did we vary the likelihood of occasional violations in the test phase so that an action was either followed by the anticipated effect most of the time (i.e., high contingency) or both effects were equally likely (i.e., no contingency). Previous research had shown that action-effect learning is critically dependent on probabilistic contingency using a training-to-test learning contingency design (Elsner & Hommel, 2004). There, the relative frequencies of presence and absence of response and effect were varied across five groups in a learning phase (Rescorla, 1967; for a review, see De Houwer & Beckers, 2002). In the test phase, high (relative to low and no) action-effect contingencies were one factor that led to significant performance benefits for acquisition-consistent relative to -inconsistent responses. Here, we argue that the goal repetition effect should be similarly susceptible to contingency learning, with a stronger continued effect of rare (high contingency) vs. frequent (no contingency) unrealized internal outcome representations per test block. Both of our research questions require a novel experimental paradigm that allows for the registration of participants’ action selection, or more precisely, goal selection, relative to both their previous response and the response of the co-actor.

To this end, we developed a novel two-step joint goal setting paradigm (inspired by Krishna & Götz, 2024; van der Wel, 2015; see also Van der Biest et al., 2024): The participant and the co-actor (a confederate) move a target relay-like from the bottom center to the top left or right corner of the computer screen in two steps. In the first step, the participant moves the target halfway to either corner via one keypress. In the second step, the co-actor moves the target to its final position. In a first learning block, the participant’s directional choice (left vs. right) was always followed by the co-actor’s congruent target movement, thus allowing for the acquisition of interindividual action effect associations. In the then following test block(s), the co-actor occasionally moved the target to the incongruent final position, i.e., the corner opposite to the participant’s choice. More precisely, in one test block, the co-actor chose the same (congruent) corner in 80% of trials (reliable co-actor, hereafter). In the other test block, the co-actor chose the same corner in only 50% of all trials (unreliable co-actor, hereafter). In all three experiments, the critical dependent variable was participants’ choice repetition rate (left or right corner) relative to the previous trial. We expect that participants would repeat their directional choice more often after incongruent trials (when the co-actor moved the target to the opposite corner) than after congruent trials (when the co-actor moved the target to the same corner). Furthermore, we predict that this goal repetition effect would be stronger with a reliable than with an unreliable co-actor.

## Power analysis and open practices

For Experiment 1, a sensitivity analysis (calculated using MorePower 6.0.4; Campbell & Thompson, 2012) revealed that a sample size of 60 participants had sufficient statistical power of 1 − β = 0.80 to detect moderate effects of η_*p*_^*2*^ = 0.12 in a mixed-factors ANOVA. For Experiments 2 and 3, we again used MorePower 6.0.4 to determine the minimum sample size. Alpha was set to 5%, intended power was set to 90%, and the effect size was taken from Experiment 1 (η_*p*_^*2*^ = 0.16). For the main effect of Goal Congruency_*N*−1_ on the Choice Repetition Rate (CRR), the power analysis suggested a minimum sample size of 48 participants. To correct for potential dropouts, we recruited a slightly larger sample size of 52 participants in Experiment 2. Note, however, that Experiments 2 and 3 have an additional within-participant factor compared to the design of Experiment 1. Moreover, participants in Experiment 2 cooperated with the same co-actor in both Reliability conditions (50% reliable vs. 80% reliable). Due to the absence of the expected effect in Experiment 2 and the outlined design limitations of Experiment 2, we decided to collect data from 60 participants in Experiment 3 to increase the chances of finding a moderate effect of Goal CongruencyN-1 on the Choice Repetition Rate (CRR). The data are available in the following OSF repository, https://osf.io/z3puh/?view_only=f4844ca74fdc46c396bc9b7d5ca3ca6f.

## Experiment 1

Experiment 1 assessed whether the (reliable) co-actor’s congruency affected participants’ goal selection in the following trial. In a first learning block, the co-actor always continued the participants’ target movement, thus establishing interindividual action effect anticipations. In a then following test block, the co-actor occasionally did not choose the same corner as the participant, thus violating participant’s action effect anticipation. Crucially, the co-actor’s reliability was varied between participants: One group collaborated with a reliable co-actor who produced congruent action effects in 80% of the trials; another group collaborated with an unreliable co-actor who produced congruent action effects in only 50% of the trials. If participants repeat their goal selection more often when the co-actor produced an incongruent effect, this would indicate that participants want to see their anticipated action effect realized (in accordance with sociomotor theory, Kunde et al., 2018). According to the contingency learning account (e.g., Elsner & Hommel, 2004), this effect should be stronger for participants collaborating with the reliable co-actor than for those collaborating with the unreliable co-actor.

## Methods

### Participants

Sixty participants (36 female, 24 male; 53 right-handed, 5 left-handed, 2 ambidextrous; *M* = 24.57 years, *SD* = 3.42, range 18–36) from the local participant pool of the University of Regensburg volunteered for partial course credit. Data collection was conducted by one female experimenter. All participants signed an informed consent at the beginning of the experiment.

### Apparatus, stimuli, and procedure

Experiment 1 was programmed using PsychoPy v3.0 and PsychoJS (Peirce et al., 2019, 2022). Due to the COVID-19 pandemic, all experiments were conducted online via the platform pavlovia.org (Bridges et al., 2020). In addition, participant and experimenter used the campus license of the University of Regensburg’s Data Center for the video-teleconferencing software “Zoom X powered by Telekom” (Zoom, 2022). At the beginning of the experiment, participant and experimenter met via Zoom. The experimenter acted as co-actor who collaborated with the participants in the online/digital joint goal-setting task (cf. Pugliese & Vesper, 2022). To that end, Zoom’s screen-sharing and remote-control features for the participants’ screen and keyboard were used. Without the experimenter’s prior knowledge, participants were randomly assigned to one of the two conditions when they clicked on the link to the online experiment.

**Fig. 1:**
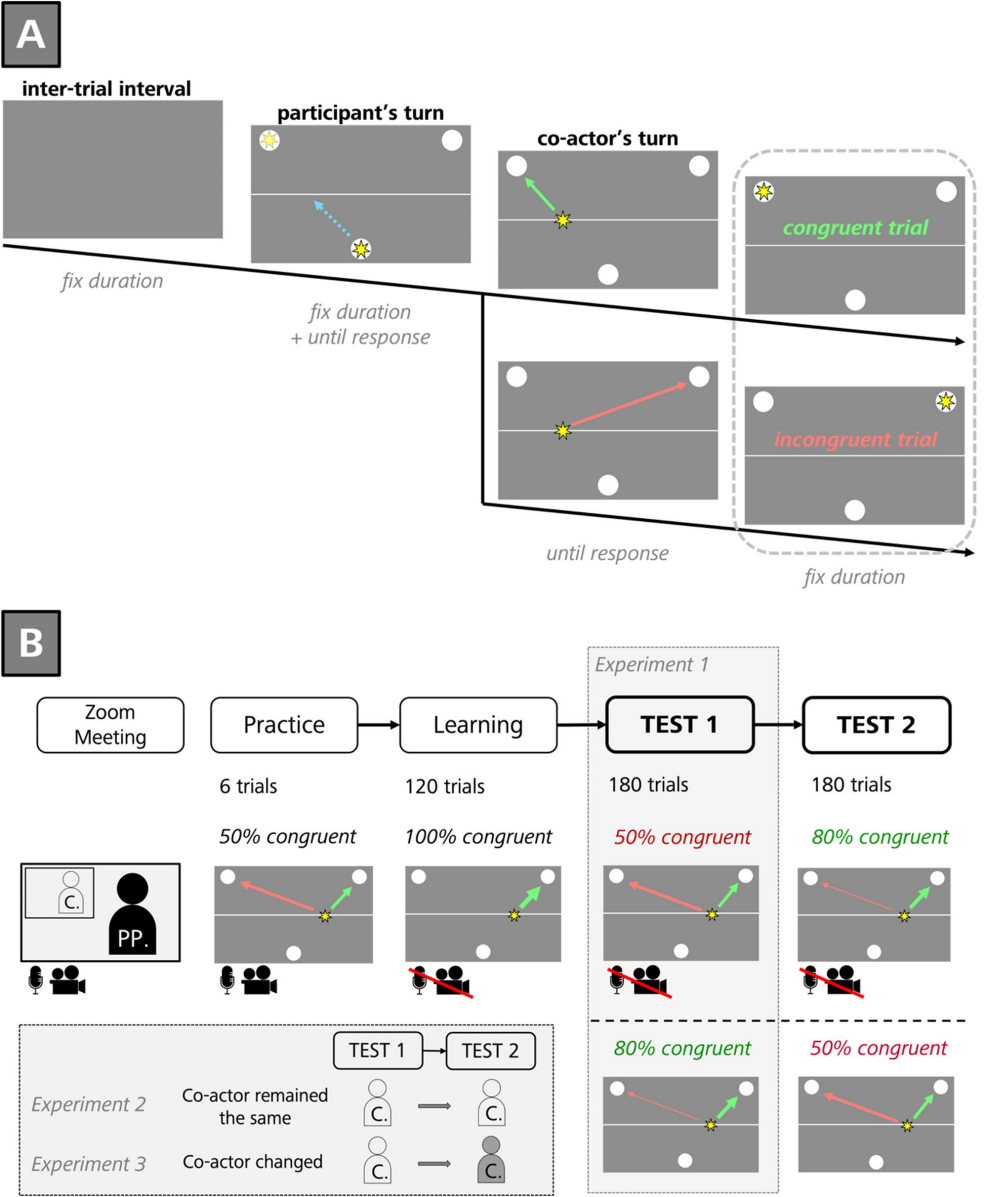
Visualization of (**A**) Trial Procedure and (**B**) the Block Procedure of Experiments 1–3. C. = Co-actor, PP. = Participant. The ‘fix duration’ was 1,000 ms in Experiment 1 and 500 ms in Experiments 2 and 3. For further details, see main text. Microsoft PowerPoint 2019 was used to create the images and arrange the panels

*Joint goal-setting task and stimuli.* In each trial, the participant and the co-actor had to navigate a star-shaped target in a relay-like manner from a starting position at the bottom center of the computer screen to one out of two possible goal circles in the top left or right corner of the screen (cf. intermittent coordination task; Krishna & Götz, 2024; see also Van der Biest et al., 2024; van der Wel, 2015). In between the bottom and top of the screen, there was a thin horizontal line that marked the stop-over position for the participant. The participants’ task was to always make the first step and move the star from the starting position halfway to either the left or right corner of the screen by pressing a key once, stopping at the midline. The experimenter then took over and moved the target to the end position (see Fig. 1, Panel A). In the instructions, participants were told that their task was to move the target together with the co-actor to one of the two upper goal circles. They were also told that the co-actor was free to follow their directional choice from the first step or not.

The start screen of each trial consisted of the bottom-starting circle (6% of the screen height), the goal circles (7,5% of the screen height), and the horizontal line (thickness: 0.5%) which were all printed in white on a dark grey background (see Fig. 1A). After 1000 ms, the instruction (font size: 4%) at the top center of the screen informed participants that it was their turn to move the target to one of the two stop-over positions on the horizontal line. Additional instructions (font size: 3%) below the two goal circles informed participants which key was associated with each of the two goal circles. Simultaneously, the star-shaped target (height and width: 5%, fill color: yellow) appeared at the start position. Participants now had to move the target to one out of the two stopover positions by pressing either the ‘X’ key (halfway to the left top corner) or the ‘M’ key (halfway to the right top corner) on their keyboard once with the index finger of their right or left hand, respectively. Simultaneously with the keypress, new instructions (font size: 4%) in the top center appeared, indicating that it was now the co-actor’s turn. Key instructions remained the same. However, unbeknownst to the participant, the co-actor’s directional ‘decision’ was determined by a list. Therefore, the co-actor always pressed the same key (key “3”) on the keyboard but counted to at least two before giving a response in order to maintain the deception. After the co-actor’s keypress the target remained in the goal circle for 500 ms before the screen turned blank for an intertrial interval of 1000 ms (see Fig. 1, Panel A).

In the learning block, the co-actor’s movement was always congruent with the participants’ choice (e.g., left midline → left end position). Critically, in the test block, the co-actor’s movement could be either congruent (same direction) or incongruent (different direction) with respect to the participant’s prior target movement. In one group (reliable co-actor), the experimenter followed the participants’ choice in 80% of all trials; in the other group (unreliable co-actor), she followed the participants’ choice in 50% of all trials (see Fig. 1, Panel B).

*Procedure.* The experiment began when participants clicked on the link provided by the experimenter via Zoom chat. They first completed a brief demographic questionnaire (sex, age, handedness; voluntary disclosures), followed by another informed consent form. If participants chose to continue by keypress, they were informed about the joint goal-setting task described above. The joint setup required participants to share both their screen and control of their keyboard with the experimenter using Zoom’s screen-sharing and remote-control features. The task was introduced with 6 practice trials in which the co-actor demonstrated his or her freedom to choose whether or not to continue the participant’s target movement. During this introductory phase of the experiment, video transmission and microphone of both the experimenter and the participants stayed turned on. When participants had no further questions, participants were asked to switch off their camera and microphone to avoid attention capture by the video transmission before the learning block started. Note that the co-actor only turned off her camera, so that the participants could still hear her keystrokes. During the break between blocks, the experimenter turned on the video transmission and asked if the participant was ok or had any questions. Before the learning and the test blocks, participants were reminded to choose their stopover position (left vs. right) spontaneously (i.e., not systematically). The learning block consisted of 120 trials, the test block of 180 trials (see Fig. 1, Panel B).

After the test blocks, participants had to rate their perceived sense of control over the target’s movements on a scale from 1 to 9 (1 = “I was in control”, 5 = “Control was fairly distributed”, 9 = “The co-actor was in control”) (inspired by (Bolt et al., 2016) by pressing the corresponding number key on their keyboard. Note that we will report the inverted ratings (such that higher numbers denote higher feelings of control). At the end of the experiment, participants additionally rated how connected they felt to the co-actor and whether they knew the co-actor in advance, each on a scale from 1 to 5. Finally, participants were debriefed and thanked for their participation.

### Design

A 2 (Goal Congruency_*N*−1_: congruent, incongruent) × 2 (Reliability: reliable, unreliable) mixed factors ANOVA was conducted. Congruency was manipulated within and the reliability was manipulated between participants.

Main dependent measure was the choice repetition rate (CRR) as a function of Goal Congruency_*N*−1_ and Reliability. We predicted that participants show a higher choice repetition rate (i.e., repeat their target choice more often) after incongruent trials than after congruent trials. This effect should be stronger with a reliable than with an unreliable co-actor. Statistically, this should result in an interaction Goal Congruency_*N*−1_ × Reliability.

## Results and discussion

### Data analysis

Only test block trials were included in the analyses. The first trial of the test block was excluded. No participants had to be excluded due to systematic responding (either switching the position on every trial or barely switching at all; our criterion was a deviation of > 3 *SD* from the mean CRR per cell of all participants), resulting in a final sample size of 60 participants.

### Choice repetition rate (CRR)

**Fig. 2:**
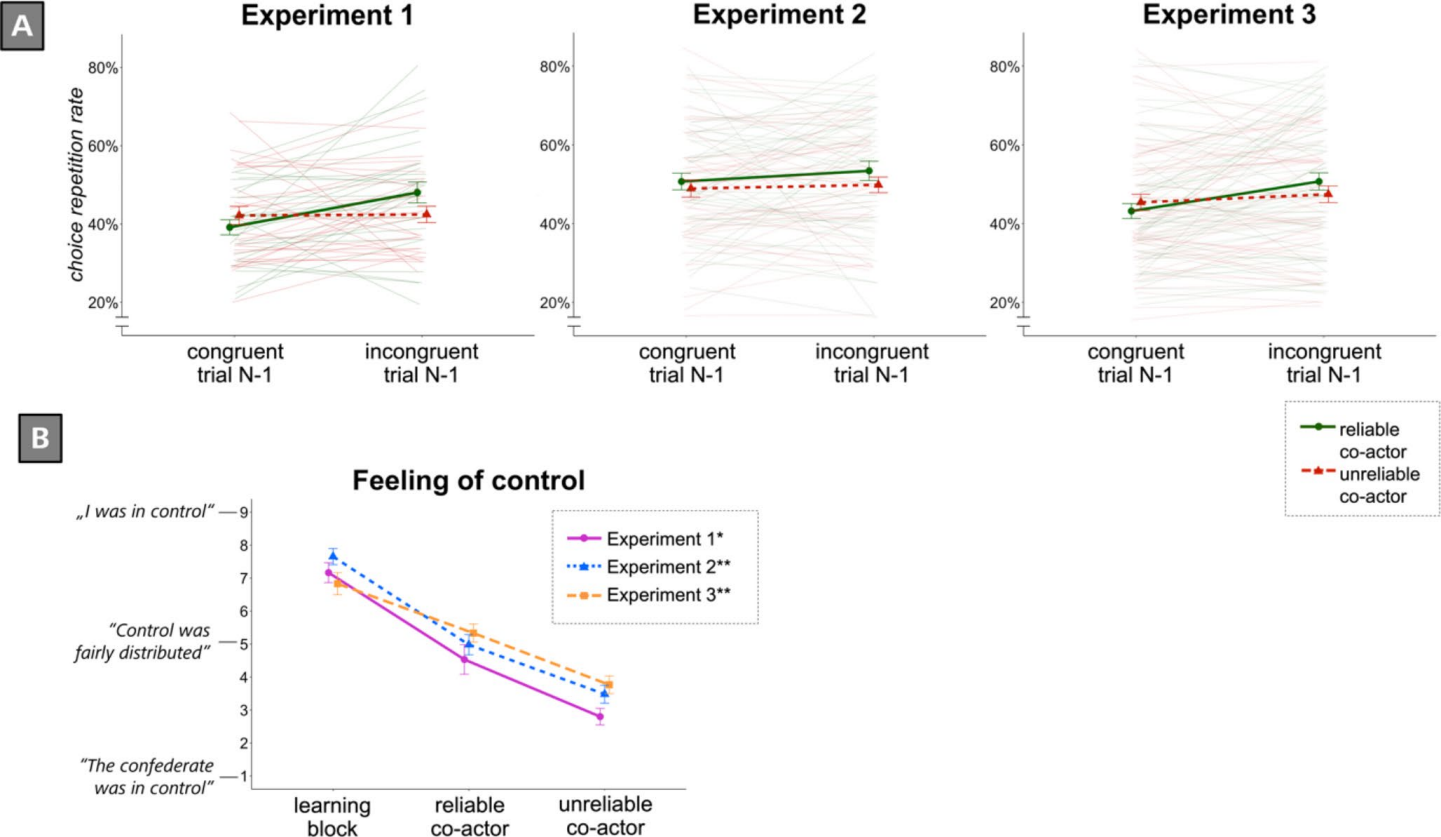
Choice Repetition Rate results of (**A**) Experiments 1, 2 and 3 as well as (**B**) Feeling of Control Ratings Experiments 1–3. Error bars indicate standard errors. In Panel A, non-transparent lines represent the group means, whereas the transparent lines represent participants’ individual data points. *Experiment 1 used a between-participants design, i.e., only half of the participants contributed data to the reliable vs. unreliable co-actor means, respectively. **Experiments 2 and 3 used a within-participant design; the order of the cooperation with a reliable vs. an unreliable co-actor was counterbalanced. RStudio was used to edit plots. Microsoft PowerPoint 2019 was used to arrange the panels

A 2 (Reliability: reliable, unreliable) × 2 (Goal Congruency_*N*−1_: congruent, incongruent) mixed-factors ANOVA revealed a significant main effect of Goal Congruency_*N*−1_, *F*(1,58) = 11.09, *p* = .002, η_*p*_^*2*^ = 0.16, BF_10_ = 13.532: The CRR following incongruent trials (*M* = 45%, *SD* = 14) was higher than the CRR following congruent trials (*M* = 39%, *SD* = 13). As predicted, the interaction between Goal Congruency_*N*−1_ and Reliability was significant, *F*(1,58) = 7.03, *p* = .010, η_*p*_^*2*^ = 0.11, BF_10_ = 4.592: The difference between the CRR following incongruent trials (*M* = 47%, *SD* = 16) and the CRR following congruent trials (*M* = 36%, *SD* = 13) for participants collaborating with a reliable co-actor was larger than the difference between the CRR following incongruent trials (*M* = 42%, *SD* = 11) and the CRR following congruent trials (*M* = 41%, *SD* = 13) for participants collaborating with an unreliable co-actor (see Fig. 2, Panel A). Subsequent t-tests showed a significant effect of Goal Congruency_*N*−1_ for participants cooperating with a reliable co-actor, *t*(29) = 3.89, *p* < .001, *d*_*z*_ = 0.71, BF_10_ = 57.693, but not for those cooperating with an unreliable co-actor, *t* < 1, BF_10_ = 0.221. These results suggest that participants were indeed more likely to repeat their choice from the previous trial after incongruent trials. In line with our a-priori predictions, this choice repetition effect was additionally modulated by the reliability of the co-actor: Participants working with the reliable co-actor showed the goal repetition effect whereas those working with the unreliable co-actor did not.

### Subjective ratings: feeling of control

For the analysis of participants’ perception of the reliability manipulation, a 2 (Reliability: reliable co-actor, unreliable co-actor) × 2 (Block: learning, test) mixed-factors ANOVA was conducted. The ANOVA revealed a significant main effect of Block, *F*(1, 58) = 111.68, *p* < .001, η_*p*_^*2*^ = 0.66: Participants felt more in control of the target’s movements in the learning block (*M* = 7.17, *SD* = 2.34) as compared to the test block (*M* = 3.67, *SD* = 2.16). The main effect of Reliability was also significant, *F*(1,58) = 4.50, *p* = .038, η_*p*_^*2*^ = 0.072: Participants collaborating with a reliable co-actor felt more in control (*M* = 5.90, *SD* = 1.96) compared to those collaborating with an unreliable co-actor (*M* = 4.93, *SD* = 1.54). In addition, there was a significant interaction effect of Reliability and Block, *F*(1,58) = 5.36, *p* = .024, η_*p*_^*2*^ = 0.09: The difference in perceived feeling of control between the learning (*M* = 7.27, *SD* = 2.12) and the test block (*M* = 4.53, *SD* = 2.46) for participants who collaborated with a reliable co-actor was smaller than the difference between the learning (*M* = 7.07, *SD* = 2.57) and the test block (*M* = 2.80, *SD* = 1.37) for participants who collaborated with an unreliable co-actor (see Fig. 2, Panel B). These results suggest that participants noticed the change in the co-actor’s behavior between the learning and the test block.

### Experiment 2

In Experiment 2, we aimed at replicating the findings of Experiment 1 in a within-participants setup. To this end, Experiment 2 consisted of one learning block with congruent trials only (i.e., collaborating with an always compliant co-actor) and two test blocks of differing (in)congruency: Participants collaborated first with a reliable (80% congruent trials) and then with an unreliable (50% congruent trials) co-actor or vice versa (order counterbalanced across participants). Thus, the co-actor’s reliability changed between test blocks whereas the co-actor remained the same. In line with sociomotor theory (Kunde et al., 2018), participants should again repeat their goal selection from the previous trial more often after incongruent choices from the co-actor. This effect should interact with the co-actor’s overall reliability in the respective test block.

## Methods

### Participants

Fifty-two participants (34 female, 18 male; 48 right-handed, 4 left-handed; *M* = 26.04 years, *SD* = 8.01, range 18–58) from the local participant pool volunteered for a monetary compensation of € 4 or course credit. Data collection was conducted by three experimenters (one male, two female). Again, all participants provided informed consent at the beginning of the experiment.

### Apparatus and procedure

Except for the following deviations, Apparatus, Procedure, and the Joint Goal-Setting Task were identical to Experiment 1. First, Experiment 2 consisted of two test blocks instead of one. The co-actor was reliable (80% congruent trials) in one block and unreliable (50% congruent trials) in the the other. Test block order was counterbalanced across participants. Note that the co-actor was not changed in-between test blocks. Second, to shorten the extension of the total duration of the experiment due to the additional test block, the inter-trial interval (blank grey screen) and the display of the startscreen at the beginning of each trial were reduced from 1,000 ms to 500 ms. Third, to reduce the monotony of the manual response, the co-actor was allowed to use two different response keys (“1”-key in addition to the “3” key). Note that the movements of the target were still controlled by a list.

### Design

A 2 (Goal Congruency_*N*−1_: congruent, incongruent) × 2 (Reliability: reliable, unreliable) × 2 (Block order: reliable-unreliable, unreliable-reliable) mixed factors ANOVA was conducted. Congruency and Reliability were manipulated within and the order between participants.

## Results and discussion

### Data analysis

Again, only test block trials were included in the analyses. The first trial of both test blocks was excluded. No participants had to be excluded due to systematic responding (> 3 *SD* from mean CRR per block of all participants), resulting in a final sample size of 52 participants.

### Choice repetition rate (CRR)

A 2 (Goal Congruency_*N*−1_: incongruent, congruent) × 2 (Reliability: reliable, unreliable) × 2 (Order: reliable-unreliable, unreliable-reliable) mixed-factors ANOVA revealed no significant effect, *F*s ≤ 1.15, *p*s ≥ 0.288 (see Table 1). Both the main effect of Goal Congruency_*N*−1_, *F* < 1, *BF*_*10*_ = 0.466, and the interaction between Goal Congruency_*N*−1_and Reliability, *F* < 1, *BF*_*10*_ = 0.215, were below the significance level of α = 0.05. Thus, the design with one co-actor that changed reliability in-between two test blocks produced no significant effect regarding participants’ corner choices from one trial to the next. Descriptively (see Table 1), only participants who started with a reliable co-actor showed a higher choice repetition rate after incongruent target movements by the co-actor.

**Table 1:** Mean choice repetition rate (SD) of experiment 2 as percentage

	overall		reliable co-actor		unreliable co-actor	
	*incongruent*	*congruent*	*incongruent*	*congruent*	*incongruent*	*congruent*
CRR (%)	54 (17)	52 (17)				
reliable first			57 (18)	52 (17)	57 (16)	53 (18)
unreliable first			52 (21)	52 (17)	49 (18)	50 (18)

### Subjective ratings: feeling of control

To analyze participants perceptions of the co-actor’s reliability, a 3 (Block: learning, reliable co-actor, unreliable co-actor) × 2 (Order: reliable-unreliable, unreliable-reliable) mixed-factors ANOVA was conducted. The ANOVA showed a significant main effect of Block, *F*(2,100) = 70.57, *p* < .001, η_*p*_^*2*^ = 0.59: Participants felt more in control of the target’s movements in the learning block (*M* = 7.65, *SD* = 1.76) as compared to the test block with the reliable co-actor (*M* = 4.98, *SD* = 2.22), and the test block with the unreliable co-actor (*M* = 3.48, *SD* = 1.96; see Fig. 2, Panel B). All other effects were not significant, *F*s < 1. These results indicate that participants noticed the changes in the co-actor’s reliability between the learning block and the two types of reliability test blocks (reliable, unreliable). That is, even though they noticed the change in reliability in the test blocks, their choice repetition rates were not significantly affected.

### Experiment 3

In Experiment 3, we tested if Experiment 2 did not replicate the findings of Experiment 1 in a within-participants setup because the co-actor stayed the same while only his/her reliability changed between test blocks. Even though the subjective ratings suggest that participants noticed the change in reliability, the reliability did not have any impact on participant’s goal choice (and neither had the congruency_*N*−1_) in Experiment 2. A potential reason might be that the subjective ratings were taken at the end of a given block, but that this change was not salient enough to exert an influence on choice during the respective second blocks. Salience appears to be critical to the role of context in learning (Bouton, 2010), and changing the co-actor is a much more salient context change than changing the congruency behavior alone. Alternatively, participants might have realized the change in reliability (as evidenced by the subjective ratings) but this alone was not enough to also change their behavior. After all, they might have made rather stable inferences about the co-actor’s intentions based on the first test block that were not corrected in the second test block (cf. Jones & Davis, 1965). Therefore, in Experiment 3, not only the reliability but also the co-actor changed between test blocks in Experiment 3. In line with sociomotor theory (Kunde et al., 2018), we expected to replicate the results from Experiment 1: Participants should show a higher choice repetition rate after incongruent trials, especially so when interacting with a reliable co-actor.

## Methods

### Participants

Sixty participants (41 female, 19 male; 50 right-handed, 6 left-handed, 4 ambidextrous; *M* = 24.62 years, *SD* = 5.29, range 18–53) from the local participant pool volunteered for a monetary compensation of € 4 or course credit. Three experimenters were involved in data collection (two female, one male). All participants provided informed consent at the beginning of the experiment.

### Apparatus, procedure, and design

Experiment 3 was almost identical to Experiment 2. The only difference was that the co-actor changed between the two test blocks. To that end, a second experimenter joined the Zoom Meeting and the first left after a brief chat.

## Results and discussion

### Data analysis

Again, only trials of the test block were included in the analyses. The first trial of both test blocks was excluded. No participants had to be excluded due to systematic responding (> 3 *SD* from mean CRR per block of all participants), resulting in a final sample size of 60 participants for Experiment 3.

### Choice repetition rate (CRR)

A 2 (Goal Congruency_*N*−1_: incongruent, congruent) × 2 (Reliability: reliable, unreliable) × 2 (Order: reliable-unreliable, unreliable-reliable) mixed-factors ANOVA revealed a significant main effect of Goal Congruency_*N*−1_, *F* (1,58) = 11.85, *p* = .001, η_*p*_^*2*^ = 0.17, BF_10_ = 29.183: The CRR following incongruent trials (*M* = 49%, *SD* = 16) was higher than the CRR following congruent trials (*M* = 45%, *SD* = 15). Furthermore, the interaction between Goal Congruency_*N*−1_ and Reliability was significant, *F* (1,58) = 7.23, *p* = .009, η_*p*_^*2*^ = 0.11, BF_10_ = 5.962: The difference between the CRR following incongruent trials (*M* = 51%, *SD* = 18) and the CRR following congruent trials (*M* = 44%, *SD* = 15) for participants collaborating with a reliable co-actor was larger than the difference of the CRR following incongruent trials (*M* = 48%, *SD* = 17) and the CRR following congruent trials (*M* = 46%, *SD* = 16) for participants collaborating with an unreliable co-actor (see Fig. 2, Panel A). All other effects were not significant, *F*s ≤ 1.08, *p*s ≥ 0.304. Subsequent t-tests showed a significant effect of GoalCongruency_*N*−1_ for participants cooperating with a reliable co-actor, *t*(59) = 4.05, *p* < .001, *d*_*z*_ = 0.52, BF_10_ = 150.065, but not for those cooperating with an unreliable co-actor, *t*(59) = 1.30, *p* = .198, *d*_*z*_ = 0.17, BF_10_ = 0.315. Like in Experiment 1, these results show that participants were again more likely to repeat their choice from the previous trial after incongruent trials. Moreover, this choice repetition effect was further modulated by the reliability of the respective co-actor: Participants showed the goal repetition effect in the test block with the reliable co-actor, but not in the test block with the other, unreliable co-actor.

**Table 2:** Mean choice repetition rate (SD) of experiment 3 as percentage

	overall		reliable co-actor		unreliable co-actor	
	*incongruent*	*congruent*	*incongruent*	*congruent*	*incongruent*	*congruent*
CRR (%)	49 (16)	45 (15)				
reliable first			51 (20)	45 (18)	49 (18)	46 (16)
unreliable first			52 (15)	43 (13)	47 (16)	46 (17)

### Subjective ratings: feeling of control

A 3 (Block: learning, reliable co-actor, unreliable co-actor) × 2 (Order: reliable-unreliable, unreliable-reliable) mixed-factors ANOVA showed a significant main effect of Block, *F*(2,116) = 31.38, *p* < .001, η_*p*_^*2*^ = 0.35: Participants felt more in control of the target’s movements in the learning (*M* = 6.83, *SD* = 2.60) as compared to the test block in which the co-actor was reliable (*M* = 5.33, *SD* = 2.12), and the test block in which the co-actor was unreliable (*M* = 3.77, *SD* = 2.08; see Fig. 2, Panel B). All other effects were not significant, *F*s < 1. These results demonstrate that participants noticed the changes in the first co-actor’s reliability between the baseline block and the first test block, as well as the differences with respect to the second co-actor’s target movements in the second test block.

### General discussion

Sociomotor theory applies ideomotor ideas to social contexts when it suggests that one’s actions can also be represented in terms of the effects they elicit from others, resulting in anticipation of *social* action effects (Kunde et al., 2018). Applying sociomotor theory to a joint action setting (Sebanz et al., 2006), we investigated individual’s goal selection when a co-actor’s (a confederate) actions had violated the former’s action-effect anticipation. In addition, we manipulated how often the co-actor violated the participant’s action-effect anticipation per block, thus creating contexts varying in predictability (and/or contingency; cf. Elsner & Hommel, 2004). In a novel joint goal-setting paradigm, the co-actor first always continued the participant’s target movement (100% congruent trials). In the following test block(s), the co-actor’s compliance with the participants’ directional choices varied (50% vs. 80% of congruent trials). In Experiment 1 (consisting of one test block), participants repeated their corner choices more often after incongruent trials. Critically, this goal repetition effect was only present for participants collaborating with a (mostly) reliable co-actor (80% congruent trials), but not for those collaborating with an unreliable co-actor (50% congruent trials; between-participants design). In Experiment 2, participants collaborated with a co-actor who changed his reliability between two test blocks (order counterbalanced across participants). Here, the results of Experiment 1 could not be replicated. Experiment 3 was almost identical to Experiment 2, but – to make the change in reliability more salient - the co-actor changed in addition to his/her reliability in-between test blocks. As a result, we again found a higher choice repetition rate after incongruent trials, but only when interacting with a reliable co-actor. Subjective ratings of participants’ feeling of control regarding the movements of the target indicated that participants felt most in control in the learning block and were able to differentiate between a reliable and an unreliable co-actor across all three experiments.

The study presented here showed, for the first time, how action effects produced by a co-actor can systematically alter action selection in a novel joint action paradigm. Here, participants in an acquisition phase first learned action effect associations between their own action (left or right corner choice) and the spatially congruent effect produced by the co-actor (who always moved the star to the congruent corner). In the then following test phase, these action effect-anticipations were occasionally violated (when the co-actor moved the star to the incongruent corner). Results show that participants choice was repeated more often after expectation violations, but only when interacting with a reliable co-actor. The results allow for three different interpretations that may not be mutually exclusive. (1) In terms of sociomotor theory (Kunde et al., 2018), repeating one’s unrealized directional choice from the previous trial might have been the participant’s means of producing compliant behavior from the co-actor (like in a leader-follower dyad). That is, from this perspective, participant’s choice repetition in response to violations of expectation (after incongruent trials) were driven by the intention to change the co-actors behavior (see also Pesquita et al., 2018; Vesper et al., 2010). Alternatively (2) and in line with ideomotor-theory, the participant’s primary goal was not to change the behavior of the co-actor. Instead participants’ action selection was continuously driven by their anticipated (and occasionally formerly unfulfilled) action-effect (cf. Prinz, 1997). From this perspective, insisting on co-actor compliance might be seen as the participants’ means of trying to produce the anticipated but unrealized target movement from the previous trial. Thus, given the social context used in the paradigm, choice repetition could be either a non-social attempt at delayed goal realization or, in terms of the minimal architecture of joint action (Vesper et al., 2010), an attempt at nonverbal communication to the co-actor and/or follower (referred to there as a ‘coordination smoother’; see also Heintz & Scott-Phillips, 2022; Vesper & Richardson, 2014). Last, but not least (3), the results may also be interpreted as an indication of the Zeigarnik-effect (Goschke & Kuhl, 1993; Zeigarnik, 1927), according to which an unfulfilled action rests in a state of heightened activation and might therefore be repeated on the next occasion (cf. Fröber & Dreisbach, 2023). Note that the latter two interpretations may be independent from the presence of a social partner. However, because the effect presented here did not survive a simple change in reliability from one block to the next (Experiment 2), we are tempted to assume that the social context had an impact, be it only to make the change in reliability (or predictability) of the environment more salient. Note that it is difficult to disentangle the role of mechanisms based on actor reliability/predictability or goal completion motivations, because the co-actor’s target movement and the final target location are hardly separable in the context of the novel paradigm.^2^

The question whether the goal repetition effect demonstrated in the present research is truly social in nature also tackles current discussions about the potential non-social nature of sociomotor action control (e.g., Neszmélyi et al., 2022; Weller et al., 2019; see also Kim & Hommel, 2019). The present findings may well be replicable in a nonsocial setting, especially if participants’ choice repetition behavior is primarily directed at the outcome rather than the actions of the co-actor. For example, sociomotor studies that similarly inserted another person’s contribution into the chain from a participant’s action to its effect found no differences in sensory processing (Neszmélyi & Horváth, 2021) or performance (Müller, 2016) compared to analogous non-social setups. Moreover, since human actions are driven by the pursuit of goals^3^ (e.g., Heckhausen & Heckhausen, 2018; Hommel, 2022; McClelland, 1988), the goal repetition effect might well be interpreted in terms of non-social goal persistence (Feather, 1962; Moshontz & Hoyle, 2021; for a recent review, see Brandstätter & Bernecker, 2022). Regarding a potential role of co-representation, there is a large body of research showing that participants’ beliefs about their (non-human) partner’s intentionality are crucial for joint action effects (e.g., Ruys & Aarts, 2010; Sahaï et al., 2019; Tsai et al., 2008; for a recent review, see Miss et al., 2022). Thus, to address the social nature of the present research findings, a more promising avenue for future research might be to manipulate social characteristics of the co-actor, such as his/her friendliness or competence, which have been shown to modulate the degree of co-representation (e.g., Ruys & Aarts, 2010; Tufft, 2022) and are difficult to reconcile with non-social accounts (e.g., referential response coding; Dolk et al., 2013).

The present research contributes to ideomotor-inspired research in social settings in several ways. Methodologically, sociomotor studies had typically used a leader-follower dyad in which the participant initiates the ‘interaction’ and a co-actor responds with predetermined responses, usually by imitating or counter-imitating the participant’s actions (e.g., Pfister et al., 2013). In the present research, the actions of the participant and the co-actor were never identical, and the actions of the former only indirectly targeted the responses of the latter. Moreover, in the practice and test blocks, the participants were led to believe that the co-actor was also free to decide to which end position he wanted to move the target. So far, only few studies on sociomotor action control involved such higher level goal pursuit and (mutual) coordination between co-actors (e.g., Müller, 2020; for a recent review, see Neszmélyi et al., 2022). Regarding the dependent variable, ideomotor studies typically measure performance benefits for compatible (e.g., Kunde, 2001) or learned (e.g., Elsner & Hommel, 2001) action-effect associations in terms of response times and error rates. By contrast, in the present research, the goal repetition effect describes a systematic influence on participants’ action selection.

Naturally, the present research has limitations. For instance, participants in Experiment 2 indicated in their subjective control ratings that they understood the reliability difference of the co-actor’s target movements between one test block (80% congruent trials) and the other (50% congruent trials). The critical question is why they did not adapt their choice behavior accordingly. From a cognitive perspective, the change in reliability may not have been perceived early enough to influence goal choice (e.g., Bouton, 2010), but rather in hindsight when feelings of control were assessed. Changing the co-actor along with the reliability might have made this change more salient. From a social perspective, one might argue that participants collaborating with a co-actor that turned from unreliable to reliable did not trust his/her apparent change of mind. Instead, they might have concluded from his/her unreliability in the first test block that s/he is generally unreliable (cf. Jones & Davis, 1965). In addition, participants and experimenters were not systematically paired with respect to sex/gender, even though previous research has shown weaker joint action effects in mixed- than in same-sex dyads (Fabbri et al., 2023).

To conclude, the here introduced novel (joint) action selection paradigm has proven to be well suited to study sociomotor action effects (Kunde et al., 2018, for a recent review, see Neszmélyi et al., 2022). We have provided first evidence that if a co-actor violates one’s action effect anticipation individuals tend to repeat their goal selection, presumably in order to see their internal goals realized (cf. Prinz, 1997). This goal repetition effect, however, is critically dependent on the overall reliability of the co-actor.

## Data Availability

The data is available at the following OSF repository, https://osf.io/z3puh/?view_only=726231c57bae4087920fb98205855025.

## Footnotes

1 There is also evidence for joint response-effect compatibility, i.e. response-effect couplings *across* two co-actors (Pfister et al., 2014): In a joint setup with each co-actor controlling one response alternative, responses that would produce compatible effects in a solo setup were indeed initiated faster.

2 For instance, if we introduce a predictable condition that produces opposite effects (e.g., 20% congruent trials) with respect to the co-actor’s movements/the final target location, then the participants are again in control of the co-actor’s movements/final target location (80% contingency) and can easily adapt their action-effect anticipation. After all, previous research has shown that both movement-effect (here: the participant’s target movement and the final target location) and target-effect (here: the co-actor’s target movement and the final target location) associations contribute to the formation of action-effect associations, as long as both are contingently related to the effect (Hoffmann et al., 2009). In consequence, in an ‘opposing co-actor condition’, the rare cases in which the co-actor continues the participants’ target movements stand out as inconsistent with their ‘inverted’ (social) action-effect anticipation. Therefore, it is reasonable to assume that after these congruent cases (rather than the incongruent ones), the participants will tend to repeat their goal choice in the following trial, thus producing an ‘inverted goal repetition effect’. This prediction holds for both an underlying mechanism based on co-actor reliability/predictability (i.e., participants aim to control the co-actor’s movements) and goal completion effects (i.e., participants aim for a delayed realization of their internal outcome representation).

3 Here, goals are understood as cognitive representations of a desired state that motivate behavior to achieve that state (e.g., Austin & Vancouver, 1996).
